# Supplementary material for: Posttranslational Acylations of the Rat Brain Transketolase Discriminate the Enzyme Responses to Inhibitors of ThDP-Dependent Enzymes or Thiamine Transport
Source: Int J Mol Sci. 2024 Jan 11;25(2):917. doi: 10.3390/ijms25020917 (PMC10815635; doi:10.3390/ijms25020917)
Supplement: Supplementary file 1 [file ijms-25-00917-s001.zip › ijms-2706696-supplementary.pdf]

International Journal of
Molecular Sciences

MDPI

Article

Posttranslational Acylations of the Rat Brain Transketolase Discriminate the Enzyme Responses to Inhibitors of ThDP-Dependent Enzymes or Thiamine Transport

Vasily A. Aleshin [1,2], Thilo Kaehne [3], Maria V. Maslova [4], Anastasia V. Graf [1,4] and Victoria I. Bunik [1,2,5,*]

Supplementary data

Table S1. The peptides used for the relative quantifications by MS. The acylated (P1, P3, P5) and reference (P2, P4, P6-9) peptides of TKT, the peptides of reference proteins actin (ActB, P10-P12) and tubulin (Tubb3, P13-P15), and the peptides of sirtuin 5 (Sirt5, P16-P18) are shown. Dependent on the number of the lysine, arginine and histidine residues in a peptide, several charged precursor variants (2+, 3+ or 4+) with monoisotopic precursor mass and the C13-isotopomepic variants ([M+1] and [M+2]) are detected to increase the quantification accuracy. ox – Met oxidation (+16), cam – Cys carbamidomethylation (+57), n.a. – not analyzed.

N	Peptide	Specification	C13-isotopomepic variants of the precursors		
			monoisotopic	[M+1]	[M+2]
P1	SK(mal)DDQVTVIGAGVTLHEALAAAEM(ox)LKK	Malonyl-K499	966.5023+++	966.8366+++	967.1708+++
			725.1286++++	n.a.	n.a.
P2	SKDDQVTVIGAGVTLHEALAAAEM(ox)LKK	K499 de-malonylated	703.6285++++	703.8792++++	704.1298++++
			563.1042; +5	563.3048; +5	563.5053; +5
P3	K(ac)ISSDLDGHPVPK	Acetyl-K102	717.8830++	718.3845++	718.8858++
			478.9244+++	479.2587+++	479.5930+++
P4	ISSDLDGHPVPK	K102 deacetylated	632.8302++	633.3317++	633.8330++
			422.2226+++	422.5569+++	422.8911+++
P5	M(ox)EGYHK(ac)PDQQKLQALKDTANR	Acetyl-K6	843.7553+++	844.0896+++	844.4237+++
P6	QAFTDVATGSLGQGLGAAC(cam)GM(ox)AYTGK	TKT	1274.5940++	1275.0954++	1275.5963++
P7	LGQSDPAPLQHQVDVYQK	TKT	1012.0158++	1012.5172++	1013.0186++
			675.0130+++	675.3473+++	675.6815+++
P8	TSRPENAIIYSNNEDFQVGQAK	TKT	1241.1039++	1241.6053++	1242.1067++
			827.7383+++	828.0726+++	828.4069+++
P9	SVPM(ox)STVFYPSDGVATEK	TKT	965.9588++	966.4603++	966.9613++
P10	VAPEEHPVLLTEAPLNPK	ActB	977.5358++	978.0373++	978.5387++
			652.0263+++	652.3606+++	652.6949+++
P11	DLYANTVLSGGTTM(ox)YPGIADR	ActB	1116.0361++	1116.5375++	1117.0386++
P12	QEYDESGPSIVHR	ActB	758.8550++	759.3564++	759.8577++
			506.2391+++	506.5734+++	506.9076+++
P13	EVDEQM(ox)LAIQSK	Tubb3	703.8452++	704.3467++	704.8474++
P14	LATPTYGDLNHLVSATM(ox)SGVTTSLR	Tubb3	1311.1656++	1311.6670++	1312.1682++
			874.4462+++	874.7805+++	875.1146+++
P15	YLTVATVFR	Tubb3	535.3057++	535.8071++	536.3085++
P16	GVPVAEFNM(ox)ETTPATNR	Sirt5	925.4411++	925.9425++	926.4435++
P17	NKEPNPGHLAIAQC(cam)EAR	Sirt5	952.9734++	953.4748++	953.9758++
			635.6514+++	635.9856+++	636.3196+++
P18	VVVITQNIDELHR	Sirt5	768.4306++	768.9321++	769.4334++

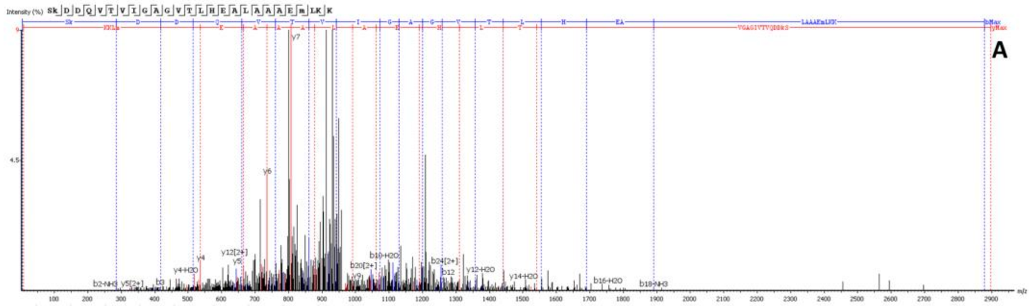

#	b	b-H2O	b-NH3	b (2+)	Seq	y	y-H2O	y-NH3	y (2+)	#
1	88.04	70.03	71.01	44.52	S					27
2	302.14	284.12	285.19	151.57	K(+86.00)	2810.46	2792.45	2793.43	1405.73	26
3	417.17	399.15	400.19	209.08	D	2596.36	2578.35	2579.34	1298.68	25
4	532.19	514.24	515.16	266.59	D	2481.34	2463.33	2464.31	1241.17	24
5	660.49	642.57	643.22	330.26	Q	2366.31	2348.30	2349.28	1183.54	23
6	759.30	741.73	742.29	380.16	V	2238.25	2220.24	2221.23	1119.63	22
7	860.51	842.35	843.68	430.68	T	2139.18	2121.17	2122.16	1070.12	21
8	959.43	941.42	941.96	480.22	V	2038.14	2020.13	2021.11	1019.57	20
9	1072.62	1054.51	1055.75	536.76	I	1939.07	1921.06	1922.04	969.79	19
10	1130.00	1111.78	1112.51	565.27	G	1825.98	1807.97	1808.96	913.49	18
11	1200.67	1182.56	1183.54	600.79	A	1768.96	1750.95	1751.94	884.54	17
12	1257.46	1239.59	1240.55	629.28	G	1697.93	1679.91	1680.90	849.46	16
13	1356.72	1338.77	1339.64	678.75	V	1640.90	1622.89	1623.88	820.95	15
14	1457.71	1439.70	1440.47	729.36	T	1541.65	1523.75	1524.81	771.63	14
15	1570.80	1552.79	1553.77	785.60	L	1440.47	1422.55	1423.76	720.89	13
16	1707.86	1689.82	1690.83	854.43	H	1327.70	1309.75	1310.68	664.39	12
17	1836.90	1818.89	1819.87	919.34	E	1190.71	1172.63	1173.67	595.82	11
18	1907.94	1889.92	1890.87	954.47	A	1061.21	1043.41	1044.61	531.35	10
19	2021.02	2003.01	2003.99	1011.01	L	990.53	972.32	973.68	496.24	9
20	2092.06	2074.05	2075.03	1046.41	A	877.70	859.47	860.51	439.24	8
21	2163.09	2145.08	2146.07	1082.04	A	806.16	788.43	789.11	403.72	7
22	2234.13	2216.12	2217.10	1117.42	A	735.43	717.43	718.38	368.20	6
23	2363.17	2345.16	2346.15	1182.56	E	664.39	646.32	647.34	332.40	5
24	2510.21	2492.20	2493.18	1255.80	M(+15.99)	535.42	517.33	518.30	268.16	4
25	2623.29	2605.28	2606.27	1312.15	L	388.29	370.28	371.26	194.65	3
26	2751.39	2733.38	2734.36	1376.02	K	275.21	257.20	258.18	138.10	2
27					K	147.11	129.10	130.09	74.06	1

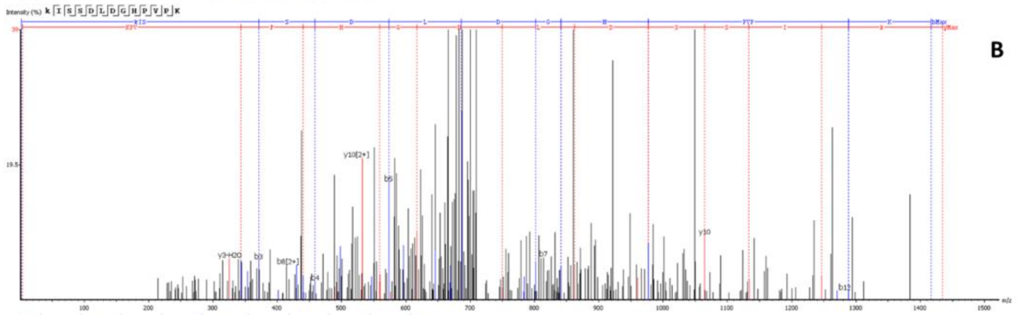

#	b	b-H2O	b-NH3	b (2+)	Seq	y	y-H2O	y-NH3	y (2+)	#
1	171.11	153.10	154.09	86.06	K(+42.01)					13
2	284.20	266.19	267.17	142.60	I	1264.65	1246.39	1247.63	632.83	12
3	371.24	353.36	354.20	186.11	S	1151.57	1133.65	1134.54	576.20	11
4	458.20	440.33	441.15	229.63	S	1064.53	1046.53	1047.51	532.61	10
5	573.59	555.28	556.26	287.14	D	977.28	959.49	960.53	489.48	9
6	686.61	668.24	669.12	343.30	L	862.39	844.47	845.45	431.74	8
7	801.45	783.31	784.48	401.41	D	749.58	731.38	732.37	375.20	7
8	858.42	840.41	841.20	430.12	G	634.37	616.36	617.27	317.68	6
9	995.48	977.28	978.45	498.01	H	577.35	559.29	560.15	289.17	5
10	1092.53	1074.52	1075.51	546.59	P	440.33	422.28	423.26	220.64	4
11	1191.60	1173.59	1174.57	596.28	V	343.30	325.44	326.21	172.12	3
12	1288.52	1270.63	1271.63	645.28	P	244.17	226.15	227.14	122.58	2
13					K	147.11	129.10	130.09	74.06	1

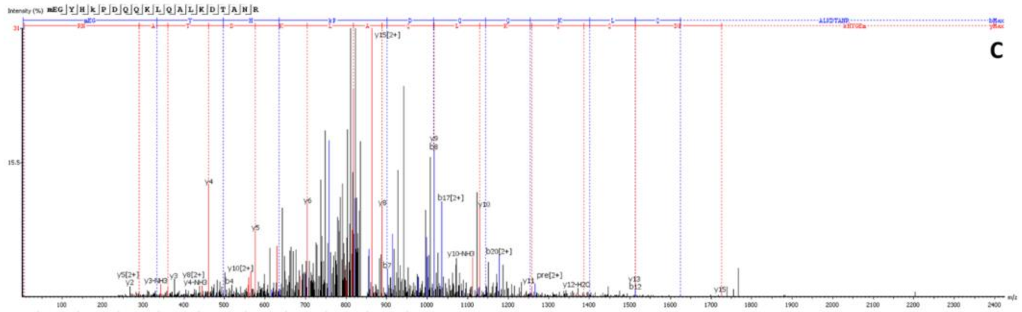

#	b	b-H2O	b-NH3	b (2+)	Seq	y	y-H2O	y-NH3	y (2+)	#
1	148.04	130.03	131.02	74.52	M(+15.99)					21
2	277.09	259.08	260.06	139.04	E	2382.22	2364.21	2365.19	1191.61	20
3	334.22	316.10	317.16	167.55	G	2253.17	2235.16	2236.15	1127.09	19
4	497.27	479.36	480.14	249.16	Y	2196.15	2178.14	2179.12	1098.58	18
5	634.39	616.53	617.33	317.16	H	2033.09	2015.08	2016.06	1017.04	17
6	804.34	786.32	787.31	403.02	K(+42.01)	1896.03	1878.02	1879.00	948.51	16
7	901.47	883.38	884.36	451.36	P	1726.08	1707.91	1708.90	863.70	15
8	1016.50	998.16	999.59	508.71	D	1628.87	1610.86	1611.84	815.01	14
9	1144.57	1126.46	1127.65	572.74	Q	1513.90	1495.83	1496.82	757.57	13
10	1272.53	1254.66	1255.50	636.55	Q	1385.78	1367.65	1368.76	693.39	12
11	1400.19	1382.62	1383.60	700.69	K	1257.79	1239.72	1240.87	629.34	11
12	1513.90	1495.70	1496.68	757.57	L	1129.66	1111.62	1112.43	565.55	10
13	1641.77	1623.76	1624.91	821.52	Q	1016.50	998.16	999.59	508.77	9
14	1712.81	1694.80	1695.78	856.91	A	888.53	870.48	871.50	444.37	8
15	1825.89	1807.88	1808.86	913.65	L	817.39	799.70	800.49	409.22	7
16	1953.99	1935.98	1936.96	977.60	K	704.41	686.36	687.40	353.14	6
17	2069.01	2051.00	2051.99	1035.25	D	576.27	558.09	559.27	289.11	5
18	2170.06	2152.05	2153.03	1085.59	T	461.23	443.08	444.37	231.12	4
19	2241.10	2223.09	2224.07	1121.31	A	360.23	342.11	343.21	180.60	3
20	2355.14	2337.13	2338.11	1177.76	N	289.11	271.15	272.03	145.08	2
21					R	175.12	157.11	158.09	88.06	1

Figure S1. Representative MS/MS spectra of the TKT peptides containing acylated residues. A – The peptide with malonylated K499 residue. B – The peptide with acetylated K102 residue. C – The peptide with acetylated K6 residue. The images of the spectra are created by PEAKS Studio Xpro. The fragment masses of the peptides indicated in the

tables below the MS/MS spectra, unambiguously determine the site of the modification, even if the second site is present in the sequence.

Table S2. The *p*-values for statistical significance of the differences between the control and experimental groups of rats shown in Figure 1. The significances estimated by the Student and Mann-Whitney tests are compared.

	Oxythiamine (OT) vs. Control		Metformin + Amprolium (M+A) vs. Control	
Test	Student	Mann-Whitney	Student	Mann-Whitney
Mal-K499	0.0002	0.0006	0.202	0.181
Sirt5	0.003	0.009	0.108	0.251
Ac-K102	0.278	0.198	0.598	0.557
Ac-K6	0.483	0.357	0.146	0.121
